# Soil Amendment With Different Maize Biochars Improves Chickpea Growth Under Different Moisture Levels by Improving Symbiotic Performance With *Mesorhizobium ciceri* and Soil Biochemical Properties to Varying Degrees

**DOI:** 10.3389/fmicb.2019.02423

**Published:** 2019-10-31

**Authors:** Dilfuza Egamberdieva, Li Li, Hua Ma, Stephan Wirth, Sonoko Dorothea Bellingrath-Kimura

**Affiliations:** ^1^Leibniz Centre for Agricultural Landscape Research, Müncheberg, Germany; ^2^Department of Microbiology, Faculty of Biology, National University of Uzbekistan, Tashkent, Uzbekistan; ^3^CAS Key Laboratory of Biogeography and Bioresource in Arid Land, Xinjiang Institute of Ecology and Geography, Ürümqi, China

**Keywords:** maize biochar, loamy sand, chickpea, rhizobia, nutrient acquisition, soil enzymes

## Abstract

Chickpea (*Cicer arietinum* L.) is an important legume originating in the Mediterranean and the Middle East and is now cultivated in several varieties throughout the world due to its high protein and fiber content as well as its potential health benefits. However, production is drastically affected by prevalent water stress in most soybean-growing regions. This study investigates the potential of biochar to affect chickpea-*Rhizobium* symbiotic performance and soil biological activity in a pot experiment. Two different biochar types were produced from maize using different pyrolysis techniques, i.e., by heating at 600°C (MBC) and by batch-wise hydrothermal carbonization at 210°C (HTC), and used as soil amendments. The plant biomass, plant nutrient concentration, nodule numbers, leghemoglobin (Lb) content, soil enzyme activities, and nutrient contents of the grown chickpeas were examined. Our results indicated that plant root and shoot biomass, the acquisition of N, P, K, and Mg, soil nutrient contents, soil alkaline and acid phosphomonoesterases, and proteases were significantly increased by HTC char application in comparison to MBC char under both well-watered and drought conditions. Furthermore, the application of both biochar types caused an increase in nodule number by 52% in well-watered and drought conditions by improving the symbiotic performance of chickpea with *Mesorhizobium ciceri*. Rhizobial inoculation combined with HTC char showed a positive effect on soil FDA activity, proteases and alkaline phosphomonoesterases under well-watered and drought conditions compared to the control or MBC char-amended soils. This concept, whereby the type of producing biochar plays a central role in the effect of the biochar, conforms to the fact that there is a link between biochar chemical and physical properties and enhanced plant nutrient acquisition, symbiotic performance and stress tolerance.

## Introduction

The long-term use of inorganic nitrogen fertilizers, or the application of such fertilizers at rates higher than the optimum, increases residual inorganic N with adverse effects such as soil degradation or the decline of soil biological health (Gong et al., 2013; Reckling et al., 2016). An alternative N resource to mitigate such drawbacks is biological nitrogen fixation by legumes, which plays a major role in sustaining or improving soil productivity (Santi et al., 2013; Egamberdieva et al., 2018). Legumes are grown in many countries of the world and are considered an important food source for human and animal nutrition (Lüscher et al., 2014; Egamberdieva et al., 2015). However, abiotic stresses such as drought and salinity threaten the growth and yield of legumes and other crops (Bodner et al., 2015; Egamberdieva et al., 2016a, 2017b; Abd Allah et al., 2017). Grain legumes are sensitive to drought stress, and the symbiotic performance of plants with rhizobia is known to be especially hindered by drought, resulting in decreased nodulation and nitrogen fixation (Bouhmouch et al., 2005; Farooq et al., 2017). Inhibition of root nodule formation in legumes can be attributed to the failure of rhizobial colonization in the rhizosphere, which indicates the susceptibility of bacterial proliferation to stress factors (Rehman and Nautiyal, 2002).

Chickpea (*Cicer arietinum* L.) is an important legume crop in many countries and is considered a functional food source, mostly due to its high protein content (17–31% protein) (Merga and Haji, 2019). Water deficit reduces chickpea growth and yield and leads to low N fixation due to the limitation of *rhizobium* survival and growth in the soil (Rahbarian et al., 2011). Furthermore, a reduction in photosynthetic pigments, CO_2_ assimilation rate, leaf water contents and disturbance in nutrient acquisition by plants were found under drought stress (Vadez et al., 2012; Siddiqui et al., 2015).

Different strategies have been developed to improve the tolerance of crops to drought, e.g., breeding for stress tolerant varieties (Witcombe et al., 2008), genetic engineering (Roy et al., 2011), and application of microbial inoculants (Hashem et al., 2016; Parray et al., 2016). Biochar amendment has also been repeatedly discussed as a technique to increase plant tolerance to various biotic and abiotic stresses (Akhtar et al., 2015). Biochar has been widely used as a soil amendment to improve soil fertility through improved water holding capacity (Yu et al., 2013; Lim et al., 2016), soil cation exchange capacity (Novak et al., 2009), nutrient retention, especially in soils deficient in organic matter content (Chan et al., 2007; Ibrahim et al., 2013), or soil microbial activities (Kolton et al., 2011; Egamberdieva et al., 2016b, 2017a; Soudek et al., 2016). Several reports have demonstrated improved plant growth of soybean (*Glycine max* L.) (Egamberdieva et al., 2016b), pepper (*Capsicum annuum* L.), tomato (*Lycopersicum esculentum* Mill.) (Graber et al., 2010), maize (Islami et al., 2011), and wheat (Alburquerque et al., 2014) after biochar amendment. Moreover, nodule number in the cases of soybean (Mete et al., 2015) and faba bean (Mohamed et al., 2017) significantly increased due to the addition of biochar to the soil, indicating improved symbiotic performance of the plant with rhizobia. Concerning soil biochemical properties, enzyme activities were studied to rate or monitor soil fertility and productivity (Dempster et al., 2012; Song et al., 2018). Several studies reported an increase in soil enzyme activity after biochar application; however, others reported a decrease in activity (Ameloot et al., 2013; Paz-Ferreiro et al., 2014). There is evidence accumulating that the response of plant growth, nutrient acquisition, soil biogeochemical processes and microbial communities to biochar depends on the type of feedstock and the thermal conditions during production (e.g., heating period and the final set point temperature) (Gul and Whalen, 2016).

According to Deenik et al. (2011), the chemical composition of biochar, especially pyrolysis conditions, plays a vital role in soil biological responses to biochar soil amendments. For example, Butnan et al. (2015) observed that plant growth was reduced in a sandy Ultisol amended with eucalyptus wood-derived biochar produced by high (800°C) temperatures, whereas biochar produced at 350°C enhanced plant growth. Moreover, the effect of biochar characteristics such as pyrolysis temperature and duration and the application rate of biochar on soil biological activities is highly variable (Paz-Ferreiro et al., 2014; Song et al., 2018).

In another study, straw gasification biochar increased the shoot and root growth of barley (*Hordeum vulgare* L.) in sandy soil compared to the addition of wood gasification biochar (Hansen et al., 2016). In addition, the interaction of biochar with environmental conditions is essential for determining any contrasting effects, which also depend on the physicochemical properties of biochar (Joseph et al., 2010). Therefore, elucidation of the effect of biochar type on plant growth, development and soil biochemical properties provides important guidance on the selection of feedstock type and production technology, which could be applied under specific environmental conditions. Only limited information is available in the literature regarding the effect of biochar types on the symbiotic performance of legume plants with rhizobia and plant growth under drought stress conditions.

We hypothesized that amending soil with biochar might alleviate drought stress in chickpea by improving its symbiotic interactions with rhizobia in a loamy, sandy soil. Moreover, considering that sandy loam has a pH value below 7, it was unknown whether using different biochar types would promote soil biological activity. We selected biochars produced from maize by two different pyrolysis techniques, i.e., heating at 600°C (MBC) and batch-wise hydrothermal carbonization at 210°C (HTC), and amended loamy, sandy soil under drought and well-watered conditions. The aim of the present study was (1) to evaluate the effect of two contrasting biochar types on chickpea growth, nutrient uptake (N, P, and K) and symbiotic performance with *Mesorhizobium ciceri* and (2) to determine the impact on soil nutrients and soil enzymes linked to carbon, nitrogen and phosphorus cycling.

## Materials and Methods

### Soil, Biochars and Plant Material

The soil used in the study was sandy loam collected from the upper horizon (0–15 cm depth) of an experimental arable field under irrigation operated by the Experimental Field Station of Leibniz Centre for Agricultural Landscape Research (ZALF), Müncheberg, Germany. The soil consists of clay and fine silt (7%), coarse and medium silt (19%), and sand (74%) and is characterized by the following properties: pH, 6.2; organic C content (0.55%), total N content (0.07%), P content (0.03%), K content (1.25%), and Mg content (0.18%).

Two biochar materials were used in this study, supplied from the Leibniz-Institut for Agrartechnik Potsdam-Bornim e.V. (ATB), Germany (Reibe et al., 2015). Both biochar materials were produced from maize using two different pyrolysis techniques: (i) heating at 600°C for 30 min (MBC) and (ii) batch-wise hydrothermal carbonization at 210°C and 23 bar for 8 h (HTC). The biochar properties are presented in Table 1. Chickpea (*Cicer arietinum* L.) seeds (var. Xalima) were obtained from the International Centre for Agricultural Research in the Dry Areas (ICARDA), Tashkent, Uzbekistan.

**TABLE 1:** Chemical characterization of chars (Reibe et al., 2015).

**Material**	**DM (%FM)**	**Ash (%DM)**	**C (%DM)**	**N (%DM)**	**P (g/kg FM)**	**K (g/kg FM)**	**pH**	**EC**
HTC	47.39	3.19	64.55	2.09	1.02	3.58	5.25	0.30
MBC	92.85	18.42	75.16	1.65	5.26	31.12	9.89	3.08

*FM, fresh matter; DM, dry matter; MBC, maize biochar; HTC, hydrochar; EC, electrical conductivity.*

### Plant Growth Experiment

The experiment was conducted in a plant growth chamber at the Leibniz Centre for Agricultural Landscape Research (ZALF), Germany. The following six treatments were set up under well-watered and drought conditions, for a total of 12 treatments:

(i) un-inoculated plants grown in soil without MBC or HTC biochar
(ii) un-inoculated plants grown in soil amended with MBC
(iii) un-inoculated plants grown in soil amended with HTC
(iv) inoculated plants with *M. ciceri* and grown in soil without MBC or HTC biochar
(v) inoculated plants with *M. ciceri* and grown in soil amended with MBC
(vi) inoculated plants with *M. ciceri* and grown in soil amended with HTC

Two different biochar types derived from maize, namely, MBC and HTC, were used as soil amendments. Pots (*d* = 0.16 m, *v* = 3016 cm^3^) were filled with 1000 g of air-dried soil mixed with crushed char (particle size < 3 mm) at a 2% (w/v) concentration. The chickpea seeds were surface-sterilized using 10% v/v NaOCl for 5 min and 70% ethanol for 5 min. Afterward, the seeds were rinsed five times with sterile distilled water and transferred to paper tissue for germination in a dark room at 25°C for 3–4 days. The bacterial strain *Mesorhizobium ciceri* HAMBI1750 was obtained from the Culture Collection of Helsinki University. The strain was grown in yeast mannitol broth (Sigma Aldrich, United States) for 4 days, 2 ml of the bacterial culture was pelleted by centrifugation (10,000 × *g* for 10 min), and the supernatant was discarded. The cell pellets were washed with 2 ml of PBS (20 mM sodium phosphate and 150 mM NaCl, pH of 7.0) and re-suspended in PBS to obtain a final bacterial density of 10^8^ CFU ml^–1^. Germinated seeds were dipped in bacterial suspension for 5 min and transferred to pots. Three seeds were sown in each pot, and after 1 week, the seedlings were thinned to one plant per pot.

Each treatment was replicated for three times and was arranged in a randomized complete block design. Only one plant was cultured in each pot for nodule and plant analysis. The soil moisture levels, i.e., drought (40% soil moisture) and well-watered (80% soil moisture) conditions, were monitored using the commercially available UMP-1 BT soil moisture sensor (Umwelt-Geräte-Technik GmbH, Müncheberg, Germany). Plants were grown for 40 days at a temperature of 24°C/16°C (day/night) and humidity of 50–60%. Well-watered and drought conditions were controlled at 80 and 40% soil moisture, respectively. At harvest, the chickpea root system was carefully removed, and soil adhering to the roots was collected and considered root-associated soil. The number of nodules (nodule size > 1 mm) was counted for each plant visually. The roots and shoots were oven-dried at 70°C for 48 h. After determination of the dry weight, the materials were ground using a mill fitted with a 1 mm screen and then sub-sampled for analysis.

### Plant and Leghemoglobin Content Analyses

After 40 days, the root and shoot dry biomass and nodule numbers were determined. The LB content of nodules was determined by the following method of Wilson and Reisenauer (1963). First, 0.5 mg of crushed and ground nodule tissue was mixed into 3 ml of Drabkin’s solution. The supernatant was transferred to a 10 ml tube after centrifugation at 500 *g* for 15 min. The nodule tissue was extracted twice more with 3 ml of Drabkin’s solution and combined with the supernatants. The volume was adjusted to 10 ml with Drabkin’s solution, mixed and centrifuged at 20,000^∗^*g* for 30 min. The assay was standardized with a freshly prepared solution of bovine hemoglobin.

### Soil Nutrients and Soil Enzyme Measurements

The carbon (C), nitrogen (N), phosphorus (P), and potassium (K) concentrations in plant tissues were analyzed with an inductively coupled plasma optical emission spectrometer (ICP-OES; iCAP 6300 Duo). The total carbon (Ct) and nitrogen (Nt) of the soil samples were determined by the dry combustion method (Nelson and Sommers, 1996) using an elemental determinator (TruSpec CNS). P, K, and magnesium (Mg) were analyzed with an ICP-OES (iCAP 6300 Duo). Acid and alkaline phosphomonoesterase activities were assayed according to Tabatabai and Bremner (1969). Briefly, 0.5 g of moist soil was placed in a 15 ml vial, and 2 ml of MUB buffer (pH of 6.5 for the assay of acid phosphatase or pH of 11 for the assay of alkaline phosphatase) and 0.5 ml of *p*-nitrophenyl phosphate substrate solution (0.05 M) were added. The soil suspension was incubated in a water bath at 37°C with 300 rpm shaking after the vial was capped. After 1 h of incubation, the vial was removed from the water bath, and 2 ml of NaOH (0.5 M), 0.5 ml of CaCl_2_ (0.5 M) and 5 ml of distilled water were added to stop the reaction. One milliliter of soil suspension was centrifuged at 6500 rpm for 5 min. The concentration of *p*-nitrophenol (*p*-NP) produced in the assays of acid and alkaline phosphomonoesterase and phosphodiesterase activities was calculated from a *p*-NP calibration curve after subtracting the absorbance of the control at 400 nm wavelength using a Lambda 2 UV-VIS spectrophotometer (Perkin Elmer) (Acosta-Martínez and Tabatabai, 2011).

Protease activity was assayed using the method described by Ladd and Butler (1972). Briefly, 0.5 g of soil was weighed into a glass vial, and 2.5 ml of phosphate buffer (0.2 M, pH of 7.0) and 0.5 ml of *N*-benzoyl-L-arginine amide (BAA) substrate solution (0.03 M) were added. The vials were capped and shaken in a water bath at 37°C with a rotary speed of 300 rpm for 1 h. After the incubation, 2 ml of KCl (2 M) was added to the vials to stop the reaction. One milliliter of the solution was pipetted into a microcentrifuge tube and centrifuged at 6000^∗^*g* for 10 min. A total of 0.5 ml of the clear supernatant was mixed with 4.5 ml of distilled water, 2.5 ml of sodium salicylate-NaOH and 1 ml of sodium dichloroisocyanide solution. The reaction mixture was incubated at room temperature for 30 min. The absorbance of the colored mixture was measured at 690 nm against a blank reagent solution in a spectrophotometer. Controls were used to follow the procedure described for the assay, with the exception that the BAA substrate was added after the incubation. The ammonium released was calculated by relating the measured absorbance at 690 nm to that of a calibration graph containing 0, 1.0, 1.5, 2.0, and 2.5 μg of NH_4_^+^–N mL^–1^.

The FDA hydrolytic activity assay was performed according to Green et al. (2006). A total of 0.5 mg of soil was added to a 50 ml vial, with the subsequent addition of 25 ml of sodium phosphate (0.06 M; pH of 7.6). Then, 0.25 ml of 4.9 mM FDA substrate solution was added to all vials. The tightly capped vials were mixed and incubated in a water bath at 37°C for 1 h. A 1 ml soil suspension was transferred to a 1.5 ml tube and centrifuged at 8000 rpm for 5 min. The clear supernatant was measured at 490 nm against a blank reagent solution in a spectrophotometer. Controls were used according to the procedure described for the assay, but 0.25 ml of acetone was added instead of the FDA substrate solution. The concentration of fluorescein released was calculated by reference to a standard curve with 0, 0.001, 0.005, 0.05 and 0.15 mg of fluorescein.

### Statistical Analysis

The data were subjected to univariate analysis using a general linear model in the statistical software package SPSS 22 (SPSS Inc., Chicago, IL, United States). Multiple comparisons between treatments were tested at the *p* < 0.05 level using Tukey’s honestly significant difference (HSD) test.

## Results

### Plant Shoot and Root Growth

#### Well-Watered Conditions

**FIGURE 1:**
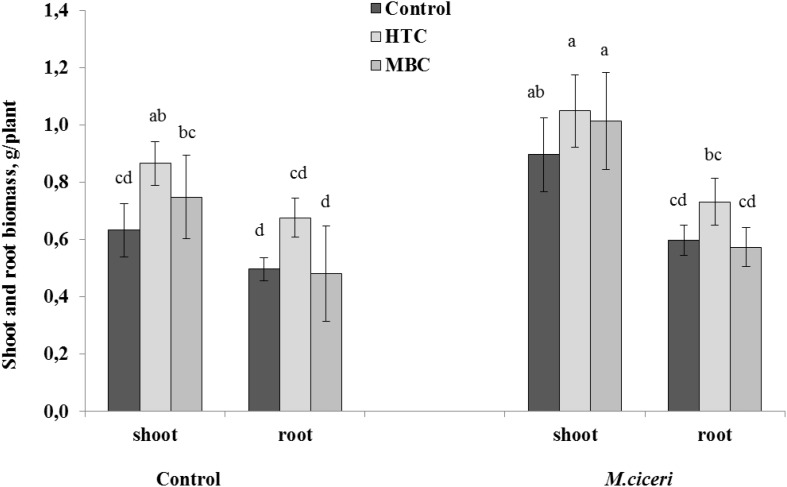
The shoot and root growth of chickpea grown in soil amended with HTC (produced from maize by batch-wise hydrothermal carbonization at 210°C) and MBC (produced from maize through pyrolysis by heating at 600°C) under well-watered conditions. The plants were un-inoculated (control) or inoculated with *M. ciceri*. Column means with different letters are significantly different based on Tukey’s HSD test at *P* < 0.05.

**FIGURE 2:**
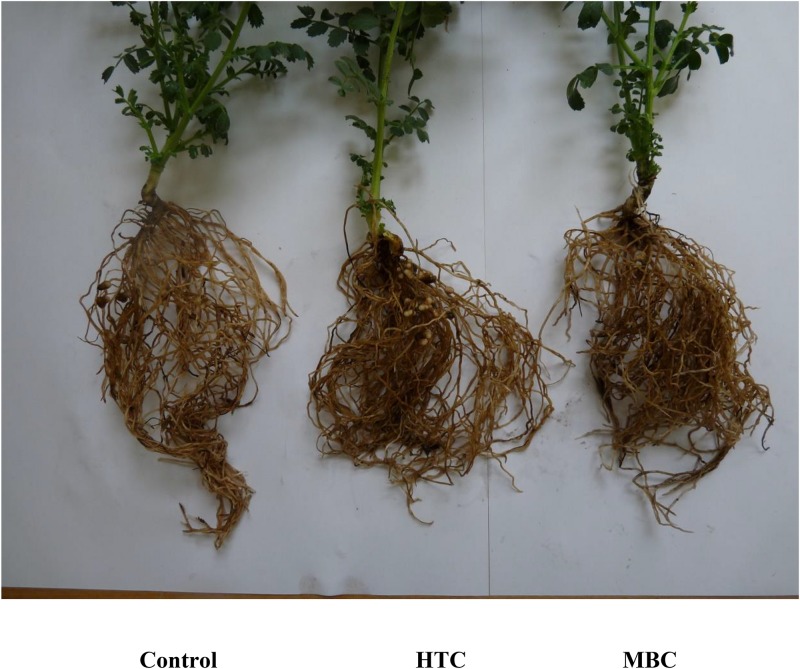
The growth of chickpea inoculated with *M. ciceri* in soil amended with 2% of HTC, MBC and without biochar (control) under the well-watered condition. Plants were grown for 40 days after the start of the experiment.

The responses of chickpea to the two biochar types were different under well-watered conditions. The growth of chickpea was significantly (*p* < 0.05) higher in soil amended with HTC than in soil without HTC addition (Figure 1). The shoot and root biomass of un-inoculated chickpea increased by 36%, whereas the shoot and root growth of inoculated plants increased by 16 and 22%, respectively. However, there were no significant effects of MBC on the growth of un-inoculated or inoculated chickpea. MBC char improved the shoot biomass of un-inoculated and inoculated chickpea by 18 and 13%, respectively, but the effect was not significant. The root biomass of neither un-inoculated nor inoculated chickpea was changed in MBC-amended soil (Figures 1,2).

#### Drought Conditions

**FIGURE 3:**
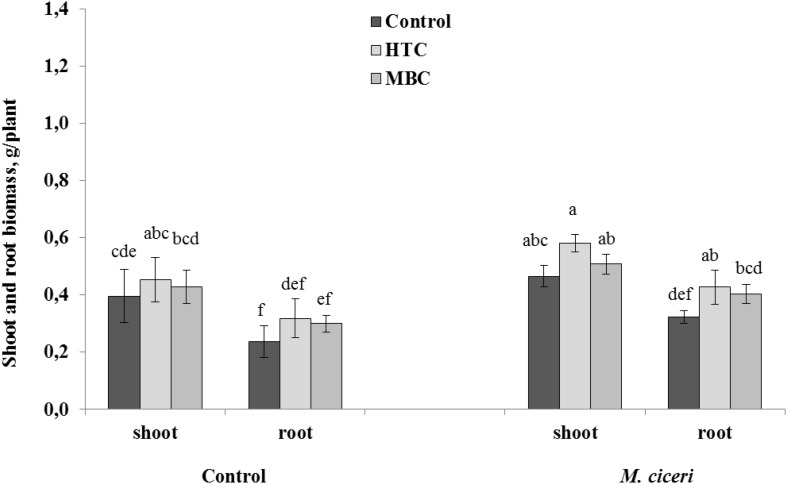
The shoot and root growth of chickpea grown in soil amended with HTC and MBC under drought conditions. The plants were un-inoculated (control) or inoculated with *M. ciceri*. Column means with different letters are significantly different based on Tukey’s HSD test at *P* < 0.05.

Overall, there were positive and significant effects of HTC on the root and shoot growth of both un-inoculated and inoculated chickpea grown under drought conditions. The soil amendment with MBC char produced more benefits to chickpea growth under drought stress than under the well-watered condition. The shoot growth was increased by 9% and root growth by 24% in both un-inoculated and inoculated plants (Figure 3). However, the stimulation was not significant when compared to control plants grown without biochar addition.

The interrelation of biochar × moisture level and biochar × inoculation showed no effects but moisture level × inoculation revealed significant interaction effects on shoot biomass (Table 2). Biochar × moisture level showed a significant interaction effect but biochar × inoculation and moisture level × inoculation showed no interaction effect on root biomass. Biochar × moisture level × inoculation showed a significant interaction effect on root biomass but not on shoot biomass.

**TABLE 2:** Interaction effects of biochar, moisture level, and inoculation on plant biomass, LB content, and plant nutrients.

**Interaction effects**	**Shoot**	**Root**	**LB**	**Shoot**	**Shoot**	**Shoot**	**Shoot**	**Shoot**	**Root**	**Root**	**Root**	**Root**	**Root**
	**biomass**	**biomass**	**content**	**C**	**N**	**P**	**K**	**Mg**	**C**	**N**	**P**	**K**	**Mg**
Biochar × Moisture level	ns	^∗∗^	ns	ns	ns	ns	^∗∗^	^∗∗^	^∗∗∗^	^∗∗^	ns	^∗∗∗^	ns
Biochar × Inoculation	ns	ns		ns	^∗∗^	^∗∗^	^∗∗^	^∗∗∗^	ns	^∗∗∗^	ns	ns	^∗∗∗^
Moisture level × Inoculation	^∗∗^	ns		ns	ns	ns	ns	^∗∗∗^	^∗^	^∗^	ns	ns	ns
Biochar × Moisture level × Inoculation	ns	^∗^		^∗∗∗^	ns	ns	ns	^∗∗^	^∗∗^	^∗∗∗^	ns	ns	^∗^

*Significance denoted by ^∗^*p* < 0.05, ^∗∗^*p* < 0.01, ^∗∗∗^*p* < 0.001.*

### Nodulation and Leghemoglobin Content

#### Well-Watered Conditions

**FIGURE 4:**
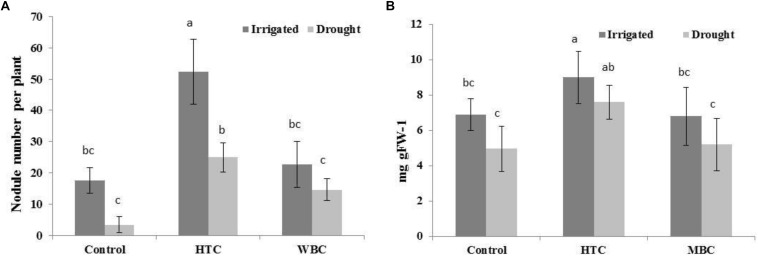
The nodule number **(A)** and LB content **(B)** of chickpea grown in soil amended with HTC and MBC under well-watered and drought conditions. The plants were inoculated with *M. ciceri*. Column means with different letters are significantly different based on Tukey’s HSD test at *P* < 0.05.

It is notable that both biochars improved the symbiotic performance of chickpea with *M. ciceri* under well-watered conditions. The nodule numbers of inoculated chickpea were 17 ± 4.01, while HTC addition to soil increased nodule numbers to 52 ± 8.1 per plant, and MBC, by 23 ± 7.2 per plant (Figure 4A). The LB content was significantly higher (9.0 mg gFW^–1^) in the nodules of plants grown in HTC-amended soil than in plants grown in control and MBC-amended soil (Figure 4B). There was no significant difference in the nodule LB content between plants grown in MBC-amended soil and control plants.

#### Drought Conditions

The nodule numbers decreased in chickpea grown under drought conditions for all variants (Figures 4A,B). The symbiotic performance of chickpea with *M. ciceri* was inhibited by drought stress, reducing nodule numbers from 17.6 to 3.4 per plant. The biochar addition increased nodule numbers to 25 ± 4.2 in HTC char-amended soil and 14.6 ± 3.1 in MBC char-amended soil, which were higher than the nodule numbers in control plants (3.4 ± 2.51) (Figure 4A).

The LB content per g of fresh weight of nodule tissue was considerably lower in the nodules of plants grown under drought conditions. The LB content in chickpea nodules was not changed in MBC-amended soil compared to control soil. However, there was a significant positive effect on the LB content in nodules of plants grown in soil with HTC addition.

The Interaction of biochar × moisture level showed no significant effect on LB content (Table 2).

### Plant Nutrient Content

#### Well-Watered Conditions

In general, the concentrations of N, P, K, and Mg in plant tissues were affected by the type of biochar and by plant growth conditions. In HTC char-amended soil, the nutrient concentration of un-inoculated and inoculated plants with *M. ciceri* was higher than that of plants grown in soil without biochar addition. Significant (*p* < 0.05) increases in N, P, K, and Mg concentrations over those in the controls were observed after HTC amendment under the well-watered conditions, with increases of 15, 35, 17, and 51%, respectively. Only K and Mg concentrations increased significantly (20–26%) in shoot tissue. The soil amendment with HTC char positively affected inoculated chickpea as well, increasing mainly N, P, and K uptake (Table 3). In MBC-amended soil, only K concentrations in the roots and shoots of un-inoculated and inoculated plants were increased compared to those in control plants grown in soil without biochar addition. The K concentration in the root tissue of un-inoculated and inoculated plants was increased by 123 and 119%, respectively, whereas that in the shoot tissue was increased by 84 and 58%. Among the nutrients, only the N and Mg concentrations in the shoots of un-inoculated plants were significantly (*p* < 0.05) increased by MBC char (Table 3).

**TABLE 3:** Carbon and nutrient concentrations (%) in chickpea shoot and root tissue grown in soils after application of HTC and MBC chars under well-watered and drought conditions.

	**Root**					**Shoot**				
	**C**	**N**	**P**	**K**	**Mg**	**C**	**N**	**P**	**K**	**Mg**
**Well-watered**										
Control	32,56^b^	1,42^c^	0,25^bc^	2,15^c^	0,94^b^	40,53^b^	1,99^b^	0,31^b^	2,13^d^	0,28^c^
*M. ciceri*	33.65^ab^	1.93^ab^	0.26^abc^	2.27^c^	1.21^ab^	44.35^a^	2.96^a^	0.30^b^	2.32^cd^	0.45^a^
HTC	35,62^a^	1,64^bc^	0,33^ab^	2,52^bc^	1,43^a^	43,35^a^	2,17^b^	0,32^b^	2,56^bc^	0,35^bc^
HTC + *M. ciceri*	35.04^ab^	2.15^a^	0.35^a^	3.34^b^	1.23^ab^	44.10^a^	3.12^a^	0.36^a^	2.69^b^	0.45^a^
MBC	32,31^b^	1,46^c^	0,25^c^	4,82^a^	0,94^b^	43,31^a^	2,08^b^	0,31^b^	3,92^a^	0,38^ab^
MBC + *M. ciceri*	34.14^ab^	1.72^ab^	0.30^abc^	4.99^a^	1.09^b^	43.55^a^	3.06^a^	0.31^b^	3.66^a^	0.42^ab^
**Drought**										
Control	27,85^c^	1,66^cd^	0,25^b^	1,99^c^	0,91^bc^	43,35^ab^	2,67^ab^	0,31^ab^	2,44^c^	0,44^c^
*M. ciceri*	25.36^c^	1.56^cd^	0.25^b^	2.00^c^	1.01^ab^	40.80^b^	3.42^a^	0.29^b^	2.62^bc^	0.49^bc^
HTC	33,86^ab^	1,53^d^	0,29^ab^	2,03^c^	1,16^a^	41,70^ab^	2,14^b^	0,31^ab^	2,81^b^	0,62^a^
HTC + *M. ciceri*	35.51^a^	2.21^a^	0.32^a^	2.45^b^	1.15^a^	44.25^a^	3.50^a^	0.35^a^	2.53^bc^	0.51^b^
MBC	33,51^ab^	1,70^c^	0,29^ab^	3,72^a^	0,72^d^	41,40^b^	3,14^a^	0,30^b^	3,74^a^	0,43^c^
MBC + *M. ciceri*	31.78^b^	1.88^b^	0.29^ab^	3.59^a^	0.78^cd^	42.54^ab^	3.18^a^	0.31^ab^	3.48^a^	0.50^b^

*Plants were grown for 40 days after the start of the experiment. The different letters indicate significant differences based on Tukey’s HSD test at *P* < 0.05.*

#### Drought Conditions

Furthermore, a positive effect of MBC char on the nutrient uptake of plants was observed under drought conditions. The soil amended with MBC showed an increased concentration of N, P, and K contents in plant roots inoculated with *M. ciceri* by 20, 16, and 79%, compared to control plants, respectively (Table 3). The highest concentration of K in roots and shoots was also observed under drought conditions, increasing by 86% for un-inoculated plants and 78% for plants inoculated with *M. ciceri*. A notable result was observed where the MBC char amendment reduced the Mg concentration in plant tissue under drought conditions. The acquisition of N, P, K, and Mg by roots and shoots was improved under drought conditions by HTC char amendment, and significant stimulation was observed in inoculated plants (Table 3). For example, the concentrations of C, N, P, K, and Mg in root tissue increased by up to 27, 33, 28, 23, and 26%, respectively.

The interaction of biochar × moisture level showed significant effects on shoot K, shoot Mg, root C, root N, and root K contents but no interaction effect on shoot C, shoot N, shoot P, root P, and root Mg content (Table 2). Biochar × inoculation showed significant interaction effects on shoot N, shoot P, shoot K, shoot Mg, root N, and root Mg content but no effect on shoot C, root C, root P, and root K content. Moisture level × inoculation showed significant interaction effects on shoot Mg, root C, and root N content but no impacts on shoot C, shoot N, shoot P, shoot K, root P, root K, and root Mg content. Furthermore, the interaction of biochar × moisture level × inoculation showed significant impacts on shoot C, shoot Mg, root C, root N, and root Mg content but no interaction effect on shoot N, shoot P, shoot K, root P, and root K contents.

### Soil Carbon and Nutrient Contents

#### Well-Watered Conditions

Table 4 shows the soil nutrient concentrations under well-watered and drought conditions with biochar treatment. The highest values of soil organic C, N, and Mg were observed in soil amended with HTC and MBC under both inoculation with *M. ciceri* and no inoculation. The lowest value was found in soil without biochar treatments. The soil organic C, N, P, K, and Mg concentrations in soil amended with HTC (inoculated with *M. ciceri* or without inoculation) were increased by up to 66, 29, 14, 44, and 44%, respectively, under well-watered conditions compared to uninoculated soil without biochar. The C, P, and Mg concentrations in the MBC-amended soil were twofold higher and the K concentration was threefold higher than those in the soil without MBC addition and inoculation with *M. ciceri* (Table 4). Our results reveal that the uptake of available P and K was significantly affected by MBC biochar amendment compared to soil without biochar and regardless of inoculation with *M. ciceri.* There were no significant differences in soil nutrients between treatments with inoculated and non-inoculated plants.

**TABLE 4:** Soil carbon and nutrient concentrations after application of HTC and MBC chars under well-watered and drought conditions.

	**Well-watered**					**Drought**				
	**C (%)**	**N (%)**	**P**	**K**	**Mg**	**C (%)**	**N (%)**	**P**	**K**	**Mg**
										
			**mg 100 g^–1^ soil**					**mg 100 g^–1^ soil**		
Control	0.89^c^	0,095^b^	7,49^c^	6,9^c^	4,67^c^	0.95^c^	0,094^c^	7,47^d^	13,52^b^	7,18^c^
*M. ciceri*	0.95^c^	0,099^b^	7,97^bc^	7,4^c^	5,38^c^	0.91^c^	0,100^c^	7,89^cd^	12,61^b^	6,54^c^
HTC	1.39^b^	0,132^a^	8,25^bc^	10,6^c^	6,68^b^	1.51^ab^	0,138^a^	8,75^b^	14,09^b^	7,48^bc^
HTC + *M. ciceri*	1.48^ab^	0,123^a^	8,55^b^	10,0^c^	6,75^b^	1.42^b^	0,138^a^	8,38^bc^	13,80^b^	7,07^c^
MBC	1.69^a^	0,129^a^	13,30^a^	27,3^b^	8,09^a^	1.75^a^	0,124^b^	12,52^a^	36,23^a^	8,48^ab^
MBC + *M. ciceri*	1.75^a^	0,128^a^	13,53^a^	32,7^a^	8,56^a^	1.71^a^	0,136^ab^	13,13^a^	38,79^a^	8,78^a^

*Plants were grown for 40 days after the start of the experiment. The different letters indicate significant differences based on Tukey’s HSD test at *P* < 0.05.*

#### Drought Conditions

The nutrient concentrations in soil without biochar amendment were similar between drought and well-watered soils, except for the K concentration, which was higher under drought conditions. Under drought conditions, both biochar amendments increased soil organic C as well as N content by up to 84 and 46% compared to soil without biochar, respectively. The P, K, and Mg concentrations in soil were not significantly affected by HTC amendment of soil; only the soil with MBC biochar showed higher contents, regardless of *M. ciceri* inoculation. Furthermore, the P concentration increased by 75%, that of K, by 186%, and that of Mg, by 22% in soil with inoculated plants and amended with MBC. The nutrient content did not differ significantly between the soil of plants inoculated with *M. ciceri* and that of uninoculated plants.

The interaction of biochar × moisture level showed significant effects on soil P, K, and Mg contents but no impacts on soil N content (Table 5). Biochar × inoculation showed significant interaction effects only on soil K content but no impacts on soil N, P, and Mg content. Moisture level × inoculation showed significant interaction effects on soil Mg content but no effects on either soil N, P, or K contents. There were no interaction effects of biochar × moisture level × inoculation on soil nutrients.

**TABLE 5:** Interaction effects of biochar, moisture level, and inoculation on soil nutrients and soil enzyme activities.

**Interaction effects**	**Soil**	**Soil**	**Soil**	**Soil**	**FDA**	**Protease**	**Alkaline phosphomonoesterase**	**Acid phosphomonoesterase**
	**N**	**P**	**K**	**Mg**		**activity**	**activity**	** activity**
Biochar × Moisture level	ns	^∗^	^∗^	^∗∗∗^	ns	ns	ns	^∗∗∗^
Biochar × Inoculation	ns	ns	^∗^	ns	ns	ns	ns	^∗^
Moisture level × Inoculation	ns	ns	ns	^∗∗^	ns	ns	ns	^∗^
Biochar × Moisture level × Inoculation	ns	ns	ns	ns	ns	ns	ns	ns

*Significance denoted by ^∗^*p* < 0.05, ^∗∗^*p* < 0.01, ^∗∗∗^*p* < 0.001.*

### Soil Enzymes

#### Well-Watered Conditions

**FIGURE 5:**
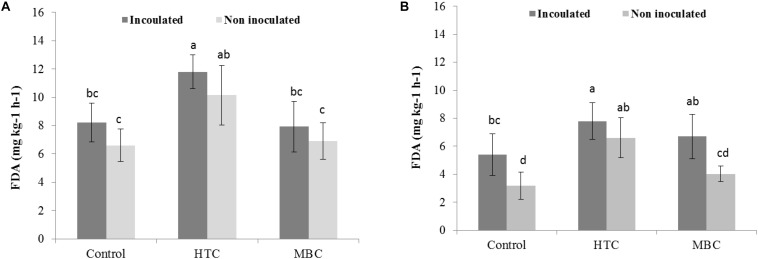
Effect of HTC and MBC on soil FDA activity under well-watered **(A)** and drought **(B)** conditions. Error bars (standard error) followed by a different letter within each column are significantly different at *P* < 0.05 based on Tukey’s HSD test.

Among the biochar treatments, adding HTC char to soil increased FDA activity in well-watered soil with inoculated and uninoculated plants by 37% (Figure 5A). The soil FDA activities did not differ between soil amended with MBC biochar and control soil without biochar (Figure 5A). The soil acid and alkaline phosphomonoesterase activities under chickpea were also affected by biochar amendment. A significant increase of 70 and 40% in acid phosphomonoesterase activity was observed under HTC and MBC amendments compared to soil without biochar, respectively. Alkaline phosphomonoesterase activity was increased by up to 30% in HTC-amended soil. Both enzyme activities were slightly but not significantly higher for chickpea inoculated with *M. ciceri* than for uninoculated controls.

**FIGURE 6:**
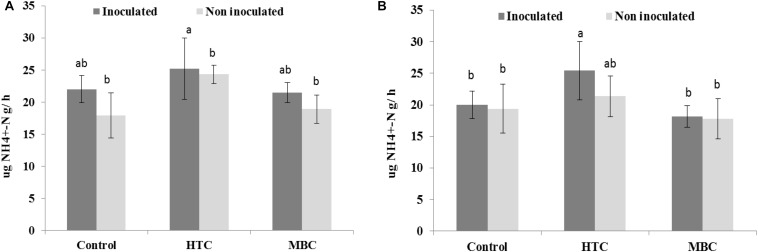
Effect of HTC and MBC on soil protease activity under well-watered **(A)** and drought **(B)** conditions. Error bars (standard error) followed by a different letter within each column are significantly different at *P* < 0.05 based on Tukey’s HSD test.

Protease activity was slightly higher in HTC-amended soil than in the control and MBC-amended soil; however, the effect was not significant (Figure 6). There was no difference in protease activity between MBC-amended soil and soil without biochar.

#### Drought Conditions

**FIGURE 7:**
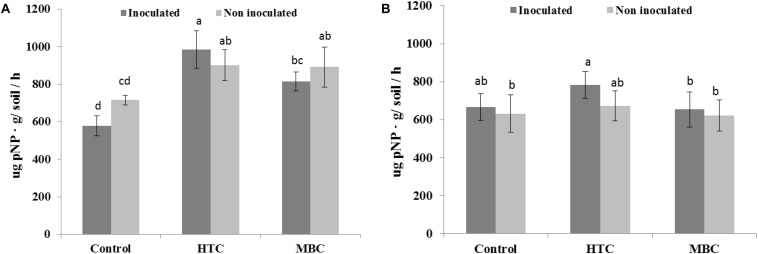
Effect of HTC and MBC on soil acid phosphomonoesterase activity under well-watered **(A)** and drought **(B)** conditions. Error bars (standard error) followed by a different letter within each column are significantly different at *P* < 0.05 based on Tukey’s HSD test.

**FIGURE 8:**
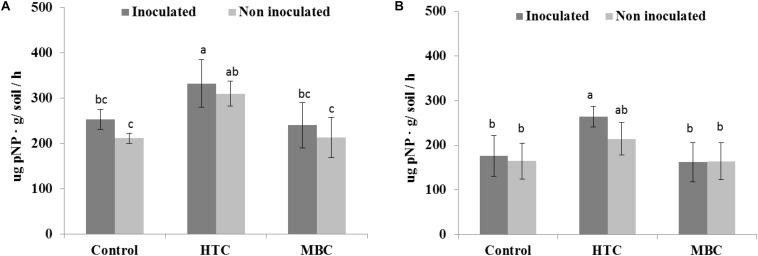
Effect of HTC and MBC on soil alkaline phosphomonoesterase activity under well-watered **(A)** and drought **(B)** conditions. Error bars (standard error) followed by a different letter within each column are significantly different at *P* < 0.05 based on Tukey’s HSD test.

Under drought conditions, soil FDA activity was higher under both biochar types, regardless of bacterial inoculation, than that of soil without biochar (Figure 5B). The HTC char amendment increased soil FDA activity by 44% and MBC by 24%, and soil FDA activity was higher with bacterial inoculation than without inoculation. Drought stress inhibited the acid and alkaline phosphomonoesterase activities of soil without biochar, regardless of inoculation with *M ciceri* (Figures 7, 8). Only HTC showed a positive effect on acid and alkaline phosphomonoesterase activities, with an increase of 17 and 49% compared to the control, but the effects were not significant for uninoculated soil (Figure 8).

There were no changes in soil protease activity under drought stress after the biochar amendments. However, an increase of 30% was observed in soil with rhizobial inoculation after HTC char amendment compared to soil without biochar (Figure 6).

The interactions of biochar × moisture level, biochar × inoculation, and biochar × inoculation showed significant impacts on soil acid phosphomonoesterase activity while biochar × moisture level × inoculation showed no interaction effect on soil acid phosphomonoesterase activity (Table 5). No interactions were found for FDA, protease, and alkaline phosphomonoesterase activities.

## Discussion

Overall, our experiment showed that the shoot and root growth, nutrient acquisition and symbiotic performance of chickpea with *M. ciceri* under well-watered and drought conditions were improved by HTC char amendment of soil. However, MBC char produced more benefits to chickpea growth under drought stress than under well-watered conditions. However, biochar and moisture level showed no significant interaction on soybean shoot biomass. It has been reported that biochar amendment increases the water holding capacity of soil (Abel et al., 2013; Bruun et al., 2014), which affects the availability of K (Rogovska et al., 2014). Joseph et al. (2010) report that the interaction of biochar with environmental conditions is an important requirement for revealing contrasting effects, which might depend on the physicochemical properties of biochar. For example, Butnan et al. (2015) observed reduced plant growth on a sandy Ultisol amended with eucalyptus wood-derived biochar produced by pyrolysis (800°C), whereas biochar produced at a lower temperature (350°C) provided higher benefits. Biochar amendment has also been shown to increase plant root and shoot growth and drought tolerance without increasing soil water availability, thus improving plant eco-physiological responses related to water status such as leaf osmotic potential, stomata resistance and water use efficiency (Kammann et al., 2011; Haider et al., 2015). Shen et al. (2016) studied the effect of two types of biochar on the plant growth and P uptake of *Lotus pedunculatus* cv barsille. The authors found that the addition of biochar from willow woodchips increased plant growth, whereas pine-based biochar did not show any positive effect on plant growth and P uptake when added to a nutrient-poor soil.

In our study, the nodule numbers and LB content were decreased in chickpea grown under drought conditions. It is notable that both biochars improved the symbiotic performance of chickpea with *M. ciceri* under both well-watered and drought conditions. Additionally, providing biochar and moisture level showed no significant interaction on LB content. According to Iijima et al. (2015), the biochar amendment of soil provides more air to nodule bacteria that adhere to the surface of biochar pores. In another study, Pietikainen et al. (2000) reported that the survival of bacteria, which were sorbed to biochar surfaces was due to the capability of biochar protecting bacteria in soil. Kolton et al. (2011) proved that soil amendment with carbon-rich biochar from citrus wood provided favorable conditions for bacterial proliferation, which increased microbial community composition in soil growing sweet peppers (*Capsicum annuum* L.). In another study, the rhizobial cell counts and nodulation of soybean were increased by the addition of biochar mixed with compost (Lehmann et al., 2011). According to Vanek and Thies (2016), the survival of *Rhizobium tropici* in biochar pores was observed over 6 months. Zahran et al. (1994) illustrated that osmotic stress might lead to an alteration in the *Rhizobium*-host plant recognition process. The severe inhibition by water deficit of root hair infection by *Rhizobium* and the formation of infection threads have also been observed (Graham, 1992; Serraj et al., 1999). In both well-watered and drought conditions, our results showed significant stimulation of root nodulation of chickpea. There are other reports on the importance of signaling factors for nodule formation impacted by biochar, e.g., Mia et al. (2014) observed stimulation of signaling for nodulation with the absorption of flavonoids and Nod factors by biochar. Wang et al. (2018) observed similar results to our findings, where soil amended with bamboo biochar that had been pyrolyzed at a temperature below 500°C stimulated root nodulation as well as soybean growth. The LB content of chickpea nodules was higher under soil amended with HTC char, but we found no distinction between MBC char and untreated control soil.

Biochar addition can induce changes in nutrient availability and may provide additional N and P (Prendergast-Miller et al., 2011) or bioavailable C sources for microbial proliferation in the rhizosphere (Zimmerman, 2010), depending on the type of biochar. For example, Shen et al. (2016) observed an increase in P uptake and plant growth by the application of biochar produced from willow woodchips compared to the non-amended soil. Soil amendment with pine-based biochar did not show any stimulatory effect on plant growth. In our study, we observed that HTC biochar increased the C content in plants, while MBC char significantly increased the K content in the roots and shoots of plants under both well-watered and drought conditions. The first indication of this positive effect was that MBC char contains higher K content than HTC char, contributing to the availability of K in soil. Wang et al. (2018) observed similar results with bamboo biochar that had been pyrolyzed at a temperature below 500°C, increasing plant growth and K uptake in soybean. In our study, biochar addition did not affect the N uptake of chickpea roots. However, the combined application of biochar with *M. ciceri* significantly increased N uptake. Corresponding results were found in a pot experiment by Rondon et al. (2007), who showed that biochar addition increased N concentrations in beans from 50% in non-biochar treatments to 72% in a biochar treatment. Furthermore, both biochar types increased the Mg uptake of chickpea roots or shoots under well-watered and drought conditions.

In our study, the addition of both types of biochar alone or combined with *M. ciceri* resulted in higher total soil N content under well-watered and drought conditions than in untreated soil, while the difference was not significant under drought conditions when amended with MBC char. In addition, no significant interaction effects among biochar, moisture level, and inoculation was found. Similar observations were reported by Han et al. (2016), where soil amendment with biochar produced from Chinese pine resulted in significant increases in soil total nitrogen. As previously reported, biochar has the capability of reducing nitrogen loss and improving nitrogen cycling in the soil (Huang et al., 2014). The mechanisms directly involved are the large surface area, highly porous structure and strong ion exchange capacity of biochars (Glaser et al., 2001), which contribute to improving the physical and chemical properties of soil and which impact soil biological activities (Anderson et al., 2014; Lentz et al., 2014). In our study, HTC char alone or combined with *M. ciceri* did not show any significant influence on soil P, K, and Mg content while the soil P, K, and Mg contents increased in the soil amended with MBC char. Nevertheless, there were significant interaction effects between biochar and moisture level on soil P, K, and Mg contents.

FDA hydrolytic activity, a measure of overall microbial activity which particularly includes esterases and protease, showed no significant distinction between MBC char and the control under well-watered conditions. However, under drought stress, soil FDA activity was higher under both biochar types than in soil without biochar. Inoculation of chickpea with *M. ciceri* increased activity under well-watered and drought conditions compared to untreated soil. In addition, no interaction effects were found among biochar, moisture level and inoculation factors on soil FDA activity. The enhancement of soil FDA hydrolytic activity by rhizobial inoculation was also observed in a 2-year field trial by Fall et al. (2016). Furthermore, increased activity of soil acid phosphomonoesterase activity was observed in HTC and MBC char treatments under well-watered conditions, whereas drought suppressed enzyme activity. However, the interaction between biochar and moisture level was pronounced for soil acid phosphomonoesterase activity. On the other hand, alkaline phosphomonoesterase activity did not change after MBC char application under either condition, whereas HTC char amendment showed a significant increase. Previous reports found increased alkaline phosphomonoesterase activities and suppression of acid phosphomonoesterase activities by application of manure-derived biochar in loamy sand soil (Jin et al., 2016). Uncertainties in the biochar effect on soil enzyme activities have also been reported (Paz-Ferreiro et al., 2014; Song et al., 2018). Wang et al. (2015) stated that the addition of maize biochar increased the activities of soil enzymes involved in C and N cycles. In our study, soil protease activity slightly increased in HTC char-amended soil and decreased in soil with MBC char amendment under drought conditions.

Rhizobial inoculation showed a positive effect on soil FDA activity, protease and alkaline phosphomonoesterases under well-watered conditions while no interaction effects were found among biochar, moisture level and inoculation. Moreover, *Rhizobia* inoculation combined with HTC char amendment improved soil enzyme activity under drought conditions compared to the control or MBC char-amended soils. These results agree with several studies reporting that *Rhizobia* might increase enzyme activity in the soil-root zone, e.g., Siczek and Lipiec (2016) found that *Rhizobium* inoculation consistently increased the activity of several enzymes in the rhizosphere.

## Conclusion

The results of our study demonstrated positive synergistic effects of biochar amendments on plant growth, plant nutrient uptake, soil nutrient contents and soil biological properties in sandy loam soil. In general, HTC char produced by batch-wise hydrothermal carbonization at 210°C had a more significant effect on the measured biological indicators than MBC char produced by pyrolysis at 600°C. This finding, that different methods of producing biochar from the same source (maize) play a critical role in the expression of soil ecological effects, underpins the assumption of a link between chemical and physical properties of biochar and enhanced plant nutrient acquisition, symbiotic performance and plant stress tolerance.

## Data Availability Statement

The raw data supporting the conclusions of this manuscript will be made available by the authors, without undue reservation, to any qualified researcher.

## Author Contributions

DE, SW, and SB-K designed the experiment. DE and HM conducted the experiment. LL, HM, and DE analyzed the data. DE, SW, and SB-K wrote the manuscript. All authors read and approved the manuscript.

## Conflict of Interest

The authors declare that the research was conducted in the absence of any commercial or financial relationships that could be construed as a potential conflict of interest.
